# Intellectual Differences Between Boys and Girls, 35 Years of Evolution in France from WISC-R to WISC-V

**DOI:** 10.3390/jintelligence12110107

**Published:** 2024-10-30

**Authors:** Jacques Grégoire

**Affiliations:** Faculty of Psychology, University of Louvain, 1348 Ottignies-Louvain-la-Neuve, Belgium; jacques.gregoire@uclouvain.be

**Keywords:** intelligence, gender, WISC-III, WISC-IV, WISC-V

## Abstract

The French adaptation of the Wechsler Intelligence Scale of Children, 5th edition (WISC-V) was an opportunity to examine if some common representations of gender differences in intellectual abilities are supported by empirical evidence. The WISC-V standardization sample provided data on a wide range of cognitive tests in a large sample of 6- to 16-year-old children representative of the French population. This sample included 517 boys and 532 girls. The WISC-V data were compared to those of the French standardization samples of three previous versions of the WISC (WISC-R, WISC-III, and WISC-IV). These four standardization samples span a 35-year period. The data analysis of the WISC-V standardization sample and the three previous versions of this intelligence scale showed that the performance gaps on intellectual tests between girls and boys have gradually narrowed over time. Almost no gender differences were observed in the WISC-V standardization sample, not only in IQ but also in key facets of intelligence. Data do not support the stereotype that girls are better at verbal tasks and boys are better at visuospatial tasks. However, some statistically significant differences remain, but the magnitude was generally small with no practical implications. The only important difference is in favor of girls and concerns performance on processing speed tasks that require visual discrimination, attentional control, and writing.

## 1. Introduction

Pierre Broca, a French neuroanatomist, adopted the first scientific approach toward quantifying intellectual differences between men and women through his research performed during the second half of the 19th century. Comparing the average weight of the brains of men and women, Broca (1861) observed a difference in weight of about 10%, heavier in the male subjects. Although, at the time, he had no objective tool to measure intelligence, Broca inferred a direct relationship between the relatively small female cerebral size and their lower average intelligence.

The first objective measures of intelligence carried out in the early 20th century did not confirm the above-mentioned assertion by Broca. Thus, Terman (1916), who adapted Binet’s intelligence test for the American population, noted a slightly superior IQ among girls aged between 5 and 13 years old, with boys being superior to girls only at the age of 14. A similar observation was made by Wechsler (1939, p. 106) using his adult intelligence test: “As our scales now stand, there are no statistically significant differences in total score between the genders, although women tend to have higher mean total scores at almost every year level”.

Some authors (e.g., Garcia 1981), however, have questioned Terman’s and Weschler’s observations, considering them artifacts as both psychometricians excluded tests that were not favorable to women, thus reducing any difference. This is confirmed by both Terman (1916) and Wechsler (1939), who admitted to having set aside certain tasks that they considered unfair for either gender. Nevertheless, this selection bias was marginal, and its impact must be put into context. Regarding the Wechsler scales, it is relevant to know that 7 of the 11 subtests of the first version of this intelligence scale, the Wechsler-Bellevue Intelligence Scale (Wechsler 1939), came from the US Army, which was used in 1917 to select the soldiers sent to the frontlines during the First World War. There is no indication that the US Army would have been concerned about gender bias in the creation of intellectual tests for the selection of men. As for the most recent versions of the Wechsler scales, the author of this article participated as a scientific adviser in their American development (WISC-IV, WAIS-IV, and WISC-V) and their French adaptation (all scales since WISC-III). He can testify that there was always an unwillingness to rule out tests that could disadvantage women. The priority for the developers has always been to select tasks that broadly cover the spectrum of intellectual abilities and best represent the major components of intelligence so that the total score on the test provides a valid measure of general intelligence.

If the battery of tests used to calculate IQ is made up of a large sample of intellectual tasks, as it is with the Wechsler scales, the overall test score allows a good estimate of the general factor (*g*), which is the central component of intelligence (Carroll 1993). Colom et al. (2000) evaluated the difference between men and women on the *g* factor from a battery of five varied intellectual tests (Vocabulary, verbal fluency, spatial rotation, inductive reasoning, and numerical aptitude). This study, conducted on a sample of 4256 women and 6219 men, showed that the difference in general intelligence between these two groups was almost zero.

While it is now well-established that, on average, men and women do not differ significantly from the point of view of their general intelligence (Giofrè et al. 2022, 2024), some authors have highlighted some possible differences that are more subtle. Feingold (1992) advanced the hypothesis that identical mean scores for men and women would mask different variances. Such a hypothesis is not new: It was already suggested in the 19th century by Ellis to try to explain the over-representation of men in the institutions for the “mentally retarded” and among the eminent intellectuals. According to this hypothesis, the distribution curve of intellectual performance for men would be more broad-based than that for women, which would lead to the over-representation of men at the two extremes of the curve, without showing any difference in the mean for each gender. A few studies have put this hypothesis to the test, with varying conclusions. Feingold (1992) presented data supporting this hypothesis based on tests of quantitative reasoning, visual analysis, spelling, and general knowledge. Larkin (2013) obtained similar results with verbal, non-verbal, and numerical reasoning tests. In contrast, on the basis of the results of a general intelligence test taken by almost all Scottish children aged 11 years, Johnson et al. (2008) observed a skewed distribution of intellectual performance where boys are primarily overrepresented at the lower levels compared to girls.

Lynn (1994) speculated that the lack of difference between men and women from the perspective of general intelligence concealed differences varying with age. No significant difference would be observed up to 14–15 years, as the earlier maturation of girls would compensate for the potential gender-based differences. On the flip side, beyond the age of 14–15 years, boys would mature further and would show a difference in general intelligence to their advantage. Lynn and Irwing (2004) confirmed this in a meta-analysis of 57 studies on the gender-based differences in performance with Raven’s Matrices. Significant differences between men and women appear in this inductive reasoning test from the age of 15. However, these differences were rather small, with around 0.25 standard deviation. Colom and Lynn (2004) and Lynn and Kanazawa (2011) reported similar results based on scores on other intelligence tests.

In addition, many authors have observed gender-based differences depending on the tasks within the batteries of tests used to measure IQ, some of them being more successfully completed by boys and others by girls. Even back in 1944, Wechsler noticed that women were often better in vocabulary tests and men in those assessing arithmetic reasoning. As a result, empirical studies multiplied and identified various tests more successfully achieved by one or the other gender. In a large meta-analysis, Hyde and Linn (1988) confirmed that girls are generally better than boys in verbal tests, at least in studies published prior to 1973. After that year, the differences converge to almost zero. (Else-Quest et al. 2010) found, in another meta-analysis, better male performances in mathematical tests. These differences, however, vary from one country to another, depending on prevailing educational practices. Several studies also investigated the gender-based differences in performance in visuospatial reasoning tests. Voyer et al. (1995) conducted a meta-analysis that shows that boys often outperform girls in such tasks. However, the degree of this superiority is variable from one test to another. It is particularly marked in timed tests for mental rotation (Voyer 2011).

Finally, several researchers have looked into the differences between boys and girls in processing speed tests. Camarata and Woodcock (2006) and Roivainen (2011) have demonstrated a very clear female superiority in these tasks. These same authors have pointed out that the same superiority is observed in reading and writing. Roivainen hypothesized that female superiority in these two areas could explain their superiority in tests of processing speed. However, the converse hypothesis is also plausible, and the causal link could not be proven. It is more likely that there is an aptitude common to both writing and tests of processing speed that explains the differences observed in the two types of activities. It could be down to fine motor skills, where girls are generally better (Peyre et al. 2019). Grégoire (2009, 2019) has put forward the hypothesis that this difference could be due to better attention control in girls than in boys. The processing speed tests, in particular that of Coding, are in fact the least successful, with children suffering from attention deficit disorders and concentration issues (Schwean and Saklofske 2005; Mayes and Calhoun 2006). Interestingly, epidemiological studies show that attention deficit and hyperactivity disorder are more common among boys than girls at a 2:1 ratio (American Psychiatric Association 2022).

The major meta-analysis by Giofrè et al. (2022), based on 79 studies of differences between school-age boys and girls on the Wechsler Intelligence Scales for Children (WISC) batteries published between 1967 and 2021, showed that most of these differences tended to narrow over time. This evolution can be interpreted within the framework of the bioecological model of Bronfenbrenner and Ceci (1994) already used to understand the Flynn effect (Grégoire et al. 2016), i.e., the improvement in average performance on intelligence tests since the 1950s in all industrialized countries (Flynn 2007). This bioecological model postulates that the genetic potentialities of individuals express themselves as a function of more or less favorable environmental factors. In the case of the Flynn effect, these environmental factors are numerous and combine to enable the actualization of the individual intellectual potential. Several of these factors could explain why, over the past 50 years, girls’ intellectual potential has been able to flourish, leading to intellectual performances close to those of boys. These factors include systematic schooling of girls from an early age, a longer school career, and a weaker impact of gender stereotypes on the choice of the school curriculum. Another factor is the reduction in family size, which leads parents to devote more time to their children, regardless of gender. The widespread use of technological tools (i.e., smartphones, PCs, video games, etc.), which are used equally by boys and girls, is also a factor that could favor the progression of intellectual skills (Fernandez-Ballesteros and Juan-Espinosa 2001).

The Wechsler scales are particularly suited for studying the evolution of intellectual differences between boys and girls. They not only provide a global measure of intelligence (Full-scale IQ) but also assess several facets of intelligence that can be gender-dependent. This variety of intellectual measures provided by the Wechsler scales is very useful since several studies have shown that, beyond general intelligence, differences between girls and boys vary according to areas of intellectual functioning (Giofrè et al. 2022, 2024).

Since their conception in 1939, Wechsler scales have slowly evolved. Several subtests present in the original version are found in all subsequent versions. In some cases, items have hardly changed, such as Coding or Block Design subtests. In other cases, such as vocabulary or arithmetic subtests, the items have been largely modified to take social and cultural developments into account. In this instance, the constructors of the scales carefully guaranteed continuity from the point of view of the type of tasks and their difficulty. This relative stability of the subtests and composite scores allows for comparing the performance of cohorts over time and studying the differences in achievement of boys and girls over a long period of time.

Whereas early versions of the Wechsler scales were essentially pragmatic in their construction, more recent versions have relied increasingly on the models of intelligence structure of Carroll (1993) and Horn and Cattell (1966), combined in the integrative CHC model (McGrew 2009). This hierarchical model of intelligence includes a general factor (*g*) at the apex and nine broad intellectual abilities at the second level (Horn and Blankson 2005). In the most recent version of the Wechsler Children’s Scale (WISC-V), the test developers’ aim was to measure, in addition to the Full-scale IQ, five of these large-range intellectual abilities: fluid intelligence (Gf), crystallized intelligence (Gc), visual processing (Gv), short-term memory (Gsm), and processing speed (Gs). In the WISC-5, these facets of intelligence are measured by a specific index. This theoretical framework is proving very useful for analyzing intellectual differences between girls and boys, beyond general intelligence.

The publication of the French WISC-V, which was standardized using a representative sample of the French population aged 6 to 16 years, offered a great opportunity to test the hypotheses presented above about the intellectual differences between men and women. It was also interesting to include the data collected during the standardization of the three previous versions of the Wechsler scale for Children (WISC-R, WISC-III, and WISC-IV) in this study, which covered a span of 35 years from 1981 to 2016. It was thus possible to highlight possible changes in differences over a period during which the education of girls and the place of women in society underwent significant transformations. On the basis of standardization samples of the four Wechsler intelligence scales for children, the evolution of differences between boys and girls was examined from the standpoint of general intelligence and the different facets of intelligence measured by indices and subtests. The differences in variability of the performance of boys and girls for IQ and the indices of WISC-V were also analyzed. Finally, based on the data of this last test, the age dependence of the differences between girls and boys was controlled.

## 2. Method

### 2.1. Instruments

The French standardization data for four Wechsler scales for children published over a period of 35 years were used for the current research. These four scales are the French adaptations of the corresponding American scales. These are:

- Wechsler Scale for Children-Revised (WISC-R), published in France in 1981. It includes 11 subtests allowing each to obtain a standardized score. On this basis, three composite scores are calculated: a Full-Scale IQ, a Verbal IQ, and a Performance IQ.
- Wechsler Scale for Children, 3rd Edition (WISC-III), published in France in 1996. It includes 13 subtests on which a Full-Scale IQ, a Verbal IQ, and a Performance IQ can be calculated. As an alternative to these last two IQs, it is possible to calculate three Indices whose composition is more homogeneous: Verbal Comprehension, Perceptual Organisation, and Processing Speed.
- Wechsler Scale for Children, 4th Edition (WISC-IV), published in France in 2005. It comprises 13 subtests that, in addition to the traditional Full-Scale IQ, are used to calculate four indices: Verbal Comprehension, Perceptual Reasoning, Working Memory, and Processing Speed.
- Wechsler Scale for Children, 5th Edition (WISC-V), published in France in 2016. It comprises 15 subtests used to calculate a Full-Scale IQ and five indices corresponding to the five factors of the second level in the CHC model (McGrew 2009): Verbal Comprehension (Gc), Visuospatial (Gv), Fluid Reasoning (Gf), Working Memory (Gsm), and Processing Speed (Gs).

From one version of the WISC to another, the Full-Scale IQ is calculated from the results of a variable set of subtests. This variation, however, does not preclude IQ comparison across the different iterations of the test. Provided that the tests used to calculate IQ have sufficient numbers and variety, the IQ estimates of the different versions of the WISC are usually close and highly correlated (Grégoire 2019). The situation is, however, different in the case of indices that are calculated on the basis of a smaller number of tasks. When these tasks are too different, the indices are not comparable from one version to another. This is the case of the Indices Perceptual Organisation (WISC-III), Perceptual Reasoning (WISC-IV), and Visual Spatial (WISC-V). The names of these Indices would suggest that they are synonymous, while in practice, these composite scores assess markedly diverse cognitive skills. Therefore, in this study, we have only chosen directly comparable scores of the following tests: Full-Scale IQ, Verbal Comprehension Index, and Processing Speed Index, as well as a number of subtests whose general characteristics are very similar across the versions (Vocabulary, Similarities, Comprehension, Information, Arithmetic, Digit Span, Letter–number Sequences, Coding, and Symbol Search). In the case of the WISC-R, only the composite scores are still available. We were therefore unable to include the subtest scores of this scale in this analysis.

### 2.2. Samples

The above-mentioned data from the French standardization of the four Wechsler scales were analyzed. The publication date, the size of the samples, and the range of ages included are listed in Table 1. In each case, it is a randomly stratified sample. The different strata and sizes were each time determined on the basis of the respective latest French general population census. The strata taken into account were age, gender, and residential geographical area. For the WISC-R, the WISC-III, and WISC-IV, the socioeconomic category of the head of the family was also taken into account. For the WISC-V, this variable was replaced by the educational level of the parents. In view of the variables taken into account and the care taken to constitute the samples, the standardization samples of the different versions of the WISC can be seen as a good representation of the 6- to 16-year-old French population.

## 3. Analysis and Results

**Figure 1:**
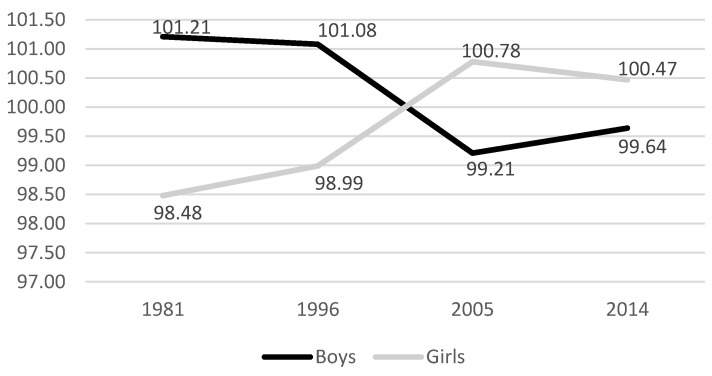
Changes in the average IQ of boys and girls over the years.

Table 2 shows the mean IQ of boys and girls from 6 to 16 years in the four analyzed versions of the WISC. In the oldest version, the WISC-R, the average IQ of boys was significantly higher than that of girls (*p* < .01). The same phenomenon was observed in the WISC-III, but the significance level reaches only *p* < .05. From the WISC-IV, the difference between the two groups was no longer statistically significant. It is important to emphasize that even when the differences were significant, their magnitude remained quite small. To be able to appreciate this magnitude, the effect sizes were calculated using Cohen’s *d*. It can thus be seen that the highest difference observed at the WISC-R corresponded to a *d* of 0.19. In other words, this difference is slightly less than one-fifth of a standard deviation. According to Cohen (1988), a *d* of 0.20 should be considered small. Figure 1 helps to visualize the changes in the average IQ of boys and girls from 1981 to 2014.

Table 3 shows the comparison of the scores of girls and boys in three Wechsler scales (WISC-III, WISC-IV, and WISC-V) for which detailed data were available. These comparisons were made for the comparable indices and subtests among the three tests. Differences between indices are expressed on a scale with a mean of 100 and a standard deviation of 15. Differences between subtests are expressed on a scale with a mean of 10 and a standard deviation of 3.

In the WISC-III, boys get significantly better results than girls in the Verbal Comprehension Index and three of the four subtests that are part of this index (Vocabulary, Comprehension, and Information). However, the effect size of these differences was small. The value of *d* for the Verbal Comprehension Index was only 0.16. In the CHC model of intelligence, this index and the related subtests are typically considered measures of crystallized intelligence (Gc), which is largely dependent on education and language acquisition. Boys’ scores were also superior on the Block Design subtest (*p* < .05; *d* = 0.13), which is a classic measure of visuospatial intelligence (Gv). They also achieve significantly higher scores on the Arithmetic subtest (*p* < .01; *d* = 0.18). What really measures this last subtest is unclear (Grégoire 2019) since it has been included in various composite scores throughout the history of the Wechsler scales. It indeed involves a set of cognitive abilities, the weight of which has varied from one version to another. The main abilities that underpin the performance in this subtest are verbal comprehension, arithmetic knowledge, attention control, and problem solving.

With the WISC-III, girls scored significantly higher than boys on the Processing Speed Index, which corresponds to processing speed (Gs) in the CHC model. The difference was statistically significant (*p* < .01) and of medium size (*d* = −0.32). This difference was observed in the two subtests that make up this index. However, it was larger for the subtest Coding (*d* = −0.37) than for the subtest Symbol Search (*d* = −0.17). These two tests measure the speed of visual analysis, attention control, associative memory, and graphomotor speed. These latter two abilities, however, play a bigger role in Coding than in Symbol Search.

With the WISC-IV, fewer higher scores for boys were observed. The differences in the subtests Information and Arithmetic remained statistically significant with *p* < .01 and a small effect size (*d* = 0.23 and 0.18). On the other hand, the performance of girls was significantly superior to that of boys in the new subtest Matrix Reasoning (*p* < .05; *d* = −0.15), which is a classic fluid reasoning test (Gf). Girls similarly scored higher than boys in the Processing Speed Index (*p* < .01; *d* = −0.30). Their performance was again the best in Coding (*p* < .01; *d* = −0.41). They also scored significantly higher than boys in the Symbol Search and the Cancellation subtests, but to a lesser extent. Cancellation is a new subtest introduced in WISC-IV that calls for the speed of perceptual analysis and demands great attention control.

In WISC-V, boys only performed significantly better than girls in two perceptual reasoning tests: Figure Weights (*p* < .01; *d* = 0.18) and Arithmetic (*p* < .05; *d* = 0.14). These subtests entail solving problems, both of which need numerical knowledge. Girls scored significantly higher than boys in the subtests Comprehension (*p* < .05; *d* = −0.15) and Picture Span (*p* < .01; *d* = 0.19). As in the previous versions of WISC, girls achieved higher performance than boys in the Processing Speed Index (*p* < .01; *d* = −0.31). This superiority recurred in the three subtests that make up this index, with a more marked advantage, as previously noted, in the Coding subtest. Interestingly, the performance of boys and girls was almost identical in the Similarities and Digit Span subtests throughout the three WISC versions.

To test the Feingold (1992) hypothesis of unequal variances of scores depending on gender, the variance of boys’ and girls’ scores on IQ and six WISC-V indices was compared using the Levene test. The results of this analysis are shown in Table 4. In contrast to Feingold’s hypothesis, the variance in the IQs of boys and girls was almost identical. As for the indices, variance differences remained low and reached a level of significance of *p* < .05 only for the Visuospatial Index where the variance of the boys’ scores was greater than that of girls’ scores. Although this difference in variance was statistically significant, it was small in magnitude, with no practical implication.

Even if the variances are equal, it is possible that the frequency of boys and girls is statistically different at the extremes of the IQ distribution. The frequency of boys and girls at the lower end (IQ ≤ 70) and the upper end (IQ ≥ 130) of the distribution is shown in Table 5. A binomial test was used to check whether the observed frequencies were statistically different from equality of frequency, i.e., 50% of boys and girls. In both cases, the binomial test was statistically non-significant. These results therefore invalidate Feingold‘s hypothesis.

To test the Lynn (1994) hypothesis of an age-dependent gender-based difference in intellectual performance, the evolution of IQ and indices differences between girls and boys across the 11 age groups of the WISC-V standardization sample was calculated.

The results of this analysis are shown in Table 6. The differences with respect to IQ were small and reached a statistical significance of *p* < .05 only at age 13, where the girls’ performances were higher than those of the boys. None of the observed results confirmed the Lynn hypothesis that around the age of 15–16 years, the trend would reverse and demonstrate superior male performances instead. In view of the evidence, no significant difference between girls and boys was observed across the 11 age groups for the indices of Verbal Comprehension, Visual Spatial, and Working Memory. A significant difference (*p* < .05) in favor of boys was observed for the Fluid Reasoning Index at the age of 10. However, it was an isolated difference with meaningless developmental implications. On the other hand, a very clear change was observed as a function of age between the girls’ and boys’ scores on the Processing Speed Index. Until the age of nine, the differences according to gender were small in magnitude and not statistically significant. From the age of 10, the size of this difference increased and reached the significance thresholds of .05 at 10 years and .01 at 13 years.

Random variations were observed from one age to another because of the rather small size of each group, but the trend was clear: girls performed significantly better than boys from 10 years of age in the Processing Speed Index. This superiority became more prominent until the age of 16. This evolution curve of the differences in the Processing Speed Index is the opposite of that expected on the basis of Lynn’s hypothesis.

## 4. Discussion

Between the French WISC-R (Wechsler 1981) and the French WISC-V (Wechsler 2016), i.e., over a period of 35 years, the average difference in IQ between boys and girls totally disappeared. This evolution is not surprising given that the lack of difference between the average IQ of men and women has been noted in developed countries for years. The amazing phenomenon is the existence of statistically significant differences in the older French standardization samples, those of the WISC-R and WISC-III. These differences were admittedly small (*d* = 0.19 and 0.14), but they were statistically significant. Unfortunately, the results of the standardization sample of the WISC-R subtests are no longer available to better understand the source of the higher male performances.

On the WISC-III, boys had superior performance in three verbal intelligence subtests (Information, Vocabulary, and Comprehension), in three visuospatial intelligence subtests (Block Design, Picture Completion, and Object Assembly) and the Arithmetic subtest. The good performances of boys were partially offset by the superior performance of girls in both processing speed subtests (Coding and Symbol Search). On one hand, male advantage in visuospatial and arithmetic tests was hardly surprising, as this superiority in both areas has often been observed in the past. On the other hand, the better performance of boys in the three verbal tests of the WISC-III was unexpected because girls have always had the reputation of being more proficient in this field. Interestingly, a similar observation was made by Pezzuti and Orsini (2016) in the standardization sample of the Italian WISC-IV. There is no evident explanation for these unexpected observations. Nevertheless, the superiority of boys in Vocabulary and Comprehension subtests disappeared in the French standardization samples of WISC-IV and WISC-V. Their superiority in the Information subtest persisted in WISC-IV but finally vanished in WISC-V. As to the superiority of boys in the Block Design subtest, it disappeared in WISC-IV and WISC-V. The two other subtests measuring visuospatial intelligence were replaced by the Visual Puzzles subtest in the WISC-V, where the difference between boys and girls was zero. The only persistent male superiority throughout the different versions of the WISC was observed in the Arithmetic subtest. Although statistically significant, the size of the difference was small (*d* = 0.14 in the WISC-V).

The lack of difference between average IQ for girls and boys was associated with an IQ distribution with almost identical variance for both genders and a frequency of boys and girls at both ends of the distribution not statistically different. The data of the French standardization of the WISC-V therefore contradicted Feingold’s (1992) hypothesis of a greater flattening of the IQ distribution curve for boys. These same standardization data also contradicted the Lynn (1994) hypothesis that an IQ difference between girls and boys would only appear at 15–16 years in favor of boys. The differences observed in WISC-V at ages 15 and 16 were instead to the advantage of girls, but they were not statistically significant. The only statistically significant difference observed in the 16-year-old group was related to the Processing Speed Index. It was large (*d* = −0.63) and favorable to girls.

While the differences between girls and boys disappeared at the level of the overall measure of intelligence, the situation was somewhat different for the major facets of intelligence found at the second level of the CHC model of intelligence. The WISC-V indices, Verbal Comprehension, Visual Spatial, Fluid Reasoning, and Working Memory measuring crystallized intelligence (Gc), visual processing (Gv), fluid reasoning (Gf), and working memory (Gsm), respectively, showed no significant gender-based difference. Significant differences, albeit small, were, however, observed in favor of boys in two tasks, Figure Weights (*d* = 0.18) and Arithmetic (*d* = 0.14), which are reliant on numerical skills. Concurrently, statistically significant, but rather small, differences in favor of girls were observed in Comprehension (*d* = 0.15) and Picture Span (*d* = 0.19).

The most striking phenomenon at this level of analysis is the difference in favor of girls on the Processing Speed Index, which is a measure of processing speed (Gs) in the CHC model of intelligence (Horn and Blankson 2005). This difference was of moderate magnitude (*d* = 0.31). Girls were significantly better in all subtests that are part of this index, particularly in Coding. This female advantage in processing speed tests was also observed in previous versions of the WISC, underlying the robustness of this finding. Similar results were reported with the standardization samples of the German WISC-IV (Goldbeck et al. 2010) and the Italian WISC-IV (Pezzuti and Orsini 2016). Several factors could explain these observations in the processing speed tasks (Camarata and Woodcock 2006; Roivainen 2011). They could be the result of better reading skills (Steinmann et al. 2023), which would promote faster processing of visual stimuli. They could also come from more precise fine motor skills and better oculomotor coordination (Cinar et al. 2023). They could finally be the result of more efficient attention control. At this point, none of these factors can be ruled out. A combination of these different factors determining the superior performance of girls in processing speed tests cannot be excluded either.

## 5. Conclusions

Based on data analysis of the standardization samples of the WISC-V and the three previous versions of this intelligence scale, it was found that the discrepancies in intellectual tests between girls and boys have gradually narrowed over time. No gender-based difference was observed in the standardization sample of the WISC-V at the IQ level, nor in most of the key facets of intelligence measured by the indices. Data from the WISC-V standardization sample do not support the stereotype that girls would be better in verbal tasks and boys in visuospatial tasks. However, some statistically significant differences remain, but they are generally small in magnitude, with no practical implication. The only larger size difference is to the advantage of the girls. It relates to performance in processing speed tasks that require significant attention control and oculomotor coordination. Apart from this difference, the standardization data of WISC-V lead to dismissing any preconception that either girls or boys are more intellectually apt at succeeding in particular fields of study or professions based on their gender.

There are, however, some limitations to the conclusions of this study. The main one concerns the tests used. Although all the versions of the WISC have been carefully developed and have high metric qualities, they included only a limited number of tests measuring the second-level components of the CHC model. The observations reported in this study should be confirmed by results obtained with other test batteries in the same population, as Giofrè et al. (2024) did for the Italian population using the Leiter-3. To better understand the nature of some differences in intellectual performance between men and women (for instance, in Coding), it would also be useful to use tasks able to reveal the cognitive processes at work in the tests where the differences were observed.

## Figures and Tables

**Table 1:** French standardization of the different versions of WISC.

Name	Year of Publication	Sample Size	Age Range
WISC-R	1981	1066	6 years 6 months to 16 years 6 months
WISC-III	1996	1120	6 years 6 months to 16 years 6 months
WISC-IV	2005	1103	6 years to 16 years 11 months
WISC-V	2014	1049	6 years to 16 years 11 months

**Table 2:** Mean IQ of boys and girls for different versions of WISC.

		Boys	Girls	Difference	*d*
WISC-R	Mean	101.21	98.48	2.73 **	0.19
	SD	15.25	15.23		
	N	533	533		
WISC-III	Mean	101.08	98.99	2.09 *	0.14
	SD	14.59	15.43		
	N	548	572		
WISC-IV	Mean	99.21	100.78	−1.57	−0.10
	SD	15.41	14.63		
	N	553	549		
WISC-V	Mean	99.64	100.47	−0.83	−0.06
	SD	14.60	14.55		
	N	517	532		

** = *p* < .01 and * = *p* < .05.

**Table 3:** Differences between the mean scores of boys and girls on the indices and the subtests of the WISC-V, WISC-IV, and WISC-III.

	WISC-V		WISC-IV		WISC-III	
	Diff.	*d*	Diff.	*d*	Diff.	*d*
*Verbal Comprehension index*	−0.44		0.06		2.35 **	0.16
Similarities	−0.02		−0.10		0.10	
Vocabulary	−0.12		0.21		0.44 *	0.14
Comprehension	−0.45 *	−0.15	−0.07		0.36 *	0.11
Information	0.30		0.74 **	0.25	0.68 **	0.23
*Visual Spatial Index*	0.54		-		-	
Block Design	−0.02		0.35		0.41 *	0.13
Visual Puzzles	0.20		-		-	
*Perceptual Reasoning index*	1.08		-		-	
Matrix Reasoning	−0.16		−0.44 *	−0.15	-	
Figure Weights	0.52 **	0.18	-		-	
Arithmetic	0.41 *	0.14	0.78 **	0.26	0.55 **	0.18
*Working Memory Index*	−1.61	-	-		-	
Digit Span	<0.01		−0.02		0.03	
Picture Span	−0.56 **	−0.19	-		-	
Letter-Number Sequences	−0.34		−0.13		-	
*Processing speed index*	−4.33 **	−0.31	−4.51 **	−0.30	−4.69 **	−0.32
Coding	−0.98 **	−0.34	−1.22 **	−0.41	−1.13 **	−0.37
Symbol Search	−0.56 **	−0.20	−0.40 *	−0.13	−0.51 **	−0.17
Cancellation	−0.39 *	−0.13	−0.43 *		-	

Composite score names are in italics and subtest names are in regular type. Negative values indicate an average score of girls higher than that of boys; ** = *p* < .01 and * = *p* < .05.

**Table 4:** Levene test of difference in variance of composite WISC-V scores for boys and girls.

	Standard Deviation	*F*	*p*
Verbal Comprehension Index	Boys = 14,798 Girls = 14,212	0.610	0.44
Visual Spatial Index	Boys = 15,418 Girls = 14,223	3.834	0.05
Fluid Reasoning Index	Boys = 15,233 Girls = 14,528	1.201	0.27
Working Memory Index	Boys = 13,852 Girls = 14,874	2.794	0.10
Processing Speed Index	Boys = 13,539 Girls = 14,354	0.846	0.36
Total IQ	Boys = 14,603 Girls = 14,551	0.004	0.951

**Table 5:** Observed frequency of boys and girls at both ends of the IQ distribution and binomial test of frequency equality.

	IQ ≤ 70	IQ ≥ 130
Boys	10	11
Girls	13	6
Binomial test	*p* = .678 (NS)	*p* = .332 (NS)

**Table 6:** Differences between boys and girls of the IQ and the five indices across the eleven age groups of the WISC-V standardization sample.

Age	N	IQ	VCI	VSI	FRI	WMI	PSI
6	101	2.24	3.82	3.03	1.62	1.94	2.03
7	100	−0.40	−2.82	−1.61	2.19	−1.95	−1.42
8	102	3.18	2.10	4.58	4.90	−1.04	0.50
9	102	−2.10	−3.86	−0.32	−0.45	0.60	−3.73
10	104	5.60	2.59	5.67	6.29 *	1.55	−6.21
11	96	−1.47	0.91	−1.60	0.24	−1.70	−4.51
12	87	−0.03	0.25	4.46	2.32	−0.60	−5.22
13	94	−5.94 *	−4.55	−3.85	−3.23	−5.00	−9.23 **
14	93	−1.96	1.06	−2.59	−1.40	−2.98	−8.27 **
15	80	−3.44	−0.79	−3.85	−2.58	−4.28	−4.66
16	90	−4.38	−4.46	1.36	0.65	−1.86	−8.50 **

Negative values indicate an average score of girls higher than that of boys; ** = *p* < .01 and * = *p* < .05.

## Data Availability

Restrictions apply to the availability of these data. Data was obtained from ECPA by Pearson, used with permission.
